# Association between Unsaturated Fatty Acid-Type Diet and Systemic Lupus Erythematosus: A Systematic Review with Meta-Analyses

**DOI:** 10.3390/nu16121974

**Published:** 2024-06-20

**Authors:** Bozhou Wang, Hanzheng Wang, Jinge Huang, Ting Zhao

**Affiliations:** 1 The First School of Clinical Medicine, Zhejiang Chinese Medical University, Hangzhou 310053, China; wbzjstc37@163.com (B.W.); brucearror@gmail.com (H.W.); 19818260586@163.com (J.H.); 2 Key Laboratory of Chinese Medicine Rheumatology of Zhejiang Province, Research Institute of Chinese Medical Clinical Foundation and Immunology, College of Basic Medical Science, Zhejiang Chinese Medical University, Hangzhou 310053, China

**Keywords:** SLE, unsaturated fatty acids, dietary management, meta-analysis

## Abstract

Background: Systemic lupus erythematosus (SLE) is a complex autoimmune disorder that affects multiple organ systems, with a higher prevalence among women in their reproductive years. The disease’s multifactorial etiology involves genetic, environmental, and hormonal components. Recent studies have highlighted the potential impact of dietary factors, particularly unsaturated fatty acids, on the modulation of SLE due to their anti-inflammatory properties. This meta-analysis aims to evaluate the association between unsaturated fatty acid consumption and the risk, progression, and clinical manifestations of SLE, providing evidence-based guidance for dietary management. Methods: We conducted a comprehensive search across major medical databases up to January 2024, focusing on studies that examined the intake of unsaturated fatty acids and the impact of such intake on SLE. Using the PICOS (population, intervention, comparator, outcomes, study design) framework, we included randomized controlled trials and case–control studies, assessing outcomes such as SLE activity, measured by SLE Disease Activity Index (SLEDAI) or the British Isles Lupus Assessment Group (BILAG) index, inflammation biomarkers. Studies were analyzed using either a fixed- or random-effects model based on heterogeneity (*I*^2^ statistic), with sensitivity analyses performed to assess the robustness of the findings. Results: Our search included 10 studies, encompassing a wide variety of designs and populations. The meta-analysis showed that a diet rich in unsaturated fatty acids is significantly associated with a reduction in SLEDAI scores (pooled SMD) of −0.36, 95% CI: −0.61 to −0.11, *p* = 0.007, indicating a beneficial effect on disease activity. Additionally, we found that unsaturated fatty acid intake has a significant impact on HDL levels, suggesting a positive effect on lipid profiles. However, no significant effects were observed on levels of the inflammatory marker IL-6 or other lipid components (LDL and cholesterol). With minimal heterogeneity among studies (*I*^2^ ≤ 15%), sensitivity analysis confirmed the stability and reliability of these results, highlighting the potential role of unsaturated fatty acids in SLE management. Conclusions: This meta-analysis suggests that dietary intake of unsaturated fatty acids may play a positive role in reducing SLE activity and may significantly affect HDL levels without having significant effects on inflammation markers or other lipid profiles. These findings support the inclusion of unsaturated fatty acids in the dietary management of SLE patients, although further research is required to refine dietary recommendations and explore the mechanisms underlying these associations.

## 1. Introduction

Systemic lupus erythematosus (SLE) is a multifaceted autoimmune disease known for its unpredictable course and diverse clinical manifestations [1,2]. It primarily affects women of childbearing age globally, presenting a significant public health challenge [2,3]. SLE can involve multiple organ systems, with symptoms ranging from mild skin lesions to severe renal damage [4]. Although the precise mechanisms underlying SLE remain largely undefined, current research indicates that environmental factors, hormonal fluctuations, and nutritional elements may promote the development of SLE in susceptible individuals by impacting cellular and humoral immune responses [5,6,7]. Managing SLE is a multifaceted task that requires a holistic consideration of the disease’s heterogeneity and individual patient differences, as well as a focus on patients’ quality of life and long-term prognosis [6,8,9]. Presently, the treatment of SLE relies primarily on pharmacological interventions such as corticosteroids and immunosuppressants, which are aimed at controlling inflammation and suppressing aberrant immune responses [8,10]. While these medications are effective, they can also have significant side effects, including increased risk of infections and osteoporosis. Recent advances have introduced new treatment options, including biologic therapies that offer targeted interventions. These newer medications have enriched the arsenal against SLE, offering more personalized treatment plans. However, the journey to find effective and less harmful treatments continues, highlighting the need for additional strategies to manage this disease.

In this context, dietary interventions emerge as a promising adjunctive strategy. Unlike pharmacological treatments, dietary changes typically carry fewer risks and can be an integral part of a comprehensive management plan. Omega-3 and omega-6 polyunsaturated fatty acids (PUFAs) [11,12,13], which are known for their exceptional anti-inflammatory properties, have become a focal point of research. As essential nutrients that the human body cannot synthesize on its own, PUFAs are essential for regulating cell signaling, maintaining cell membrane fluidity, and suppressing inflammatory processes [14]. These functions indicate that PUFAs might impact the pathogenesis of SLE by reducing the production of inflammatory mediators, modulating immune cell function, and affecting immune regulatory pathways [7,14,15]. Omega-3 and omega-6 fatty acids, while both essential, have different impacts on health. Omega-3 fatty acids generally exhibit anti-inflammatory properties and are beneficial in reducing disease activity in various inflammatory conditions, including SLE [15]. In contrast, omega-6 fatty acids can be pro-inflammatory in excessive amounts but are also necessary for normal cellular function and immune response [16,17]. The ratio between omega-3 and omega-6 fatty acids is also important; an optimal balance is believed to maximize the health benefits and minimize the risks of chronic diseases [15,18]. This hypothesis, that dietary intake of unsaturated fatty acids could affect the progression and symptoms of SLE, has sparked increasing research interest. Yet, the evidence remains inconsistent. Some studies have shown beneficial effects, suggesting that these fatty acids could modulate immune responses and inflammatory pathways, potentially alleviating disease symptoms and progression [19,20,21,22]. For instance, omega-3 fatty acids are thought to have anti-inflammatory properties, as described in [19]; they operate through various mechanisms, such as shifting the arachidonic acid pathway towards anti-inflammatory mediators and downregulating pro-inflammatory cytokines. However, other studies have yielded neutral or inconsistent results, leading to ambiguous dietary guidelines for healthcare professionals and patients [23,24].

Against the backdrop of these challenges and limitations in pharmacological treatments, the design of our systematic review and meta-analysis seeks to carefully sift through and amalgamate the existing literature to delve into the potential linkage between the intake of unsaturated fatty acids and SLE. At the heart of this initiative is the goal of conducting a thorough analysis that provides a more defined view on how various dietary practices may affect SLE risk factors, the rate of disease progression, and the clinical symptomatology in patients. Despite the growing body of research, there remains a significant gap in understanding when it comes to the precise role of different types and ratios of unsaturated fatty acids in SLE management. This study aims to address this gap by synthesizing current evidence and providing insights into optimal dietary strategies. We aspire, through this effort, to illuminate the intricacies and potential advantages of dietary adjustments in SLE management, thereby offering patients and healthcare practitioners evidence-based, tailored nutritional intervention strategies.

## 2. Methods

### 2.1. Search Strategy

The search strategy for this systematic review and meta-analysis was designed to encompass a comprehensive range of studies examining the association between unsaturated fatty acid intake and systemic lupus erythematosus (SLE). Prior to the commencement of the search, the protocol for this review was registered with the International Prospective Register of Systematic Reviews, PROSPERO (CRD42024511936) ensuring transparency and adherence to predefined objectives and methodologies. To ensure a thorough literature search, we employed an extensive array of medical and scientific databases, including PubMed, Web of science, EMBASE, and the Cochrane library. The search was tailored to include studies published up to January 2024.

The search strategy combined a series of controlled vocabulary terms and free-text words: (“unsaturated fatty acids” OR “unsaturated fatty acids” OR “monounsaturated fatty acids” OR “polyunsaturated fatty acids” OR “omega-3 fatty acids” OR “omega-6 fatty acids” OR “fish oil”) AND (“diet” OR “dietary intake” OR “diet”) AND (“systemic lupus erythematosus” OR “systemic lupus erythematosus” OR “SLE” OR “autoimmune diseases” OR “autoimmune diseases”).

### 2.2. Inclusion Criteria

In this systematic review and meta-analysis, according to PRISMA 2020 statement [25], we meticulously defined the inclusion criteria based on the PICOS framework (population, intervention, comparator, outcomes, study design) to ensure that we made a comprehensive and relevant selection of studies. The population included were participants classified with SLE, based on established diagnostic criteria such as the American College of Rheumatology (ACR) criteria [26], the Systemic Lupus International Collaborating Clinics (SLICC) criteria [27], or the 2019 American College of Rheumatology/European League Against Rheumatism (ACR/EULAR) Classification Criteria [28,29]. The primary intervention of interest was dietary intake of unsaturated fatty acids, encompassing both monounsaturated fatty acids (MUFAs) and polyunsaturated fatty acids (PUFAs), including omega-3 and omega-6 fatty acids. Comparator groups included participants who did not undergo a specific dietary intervention, those consuming different types of dietary fats (such as saturated fats), or a placebo group in supplement studies. The primary outcomes focused on were changes in SLE activity, measured using validated clinical indices such as the SLE Disease Activity Index (SLEDAI) or the British Isles Lupus Assessment Group (BILAG) index. Secondary outcomes included biomarkers of inflammation, levels of autoantibodies, and patient-reported outcome measures (PROMs) related to quality of life. Regarding study design, we included randomized controlled trials (RCTs), cohort studies, case–control studies, and cross-sectional studies.

### 2.3. Exclusion Criteria

Studies that were excluded encompassed case reports and series due to their anecdotal nature and lack of control groups, as well as editorials, commentaries, and reviews, which do not present original research. Animal studies and in vitro research were omitted to focus solely on human subjects. We excluded studies involving non-SLE populations or those with mixed autoimmune disorders where SLE-specific data were indiscernible. Regarding interventions, studies that did not specifically address the intake of unsaturated fatty acids (MUFAs and PUFAs) or lacked a clear comparator group were omitted. We also excluded studies focusing only on laboratory parameters without clinical correlation to SLE activity, or those with an inadequately short follow-up period. Non-English studies without translations and inaccessible full-text articles were excluded due to language and resource limitations. Furthermore, studies with significant methodological flaws, high risk of bias, or those that were outdated and not reflective of current medical understanding were also excluded.

### 2.4. Screening and Abstraction Process

In our systematic review and meta-analysis, we implemented a two-stage screening process to ensure the quality and relevance of the literature. Initially, two independent reviewers conducted a preliminary screening based on titles and abstracts, excluding studies unrelated to our research theme. Subsequently, those studies that passed the initial screening underwent a full-text review to further confirm their compliance with our inclusion criteria. Any discrepancies between reviewers were resolved by involving a third reviewer for discussion and consensus, ensuring the objectivity and accuracy of the assessment. Thorough data extraction was conducted for each study, covering aspects such as study design, participant characteristics (such as age and gender), specific details of interventions, control conditions, and outcome measures related to the association between unsaturated fatty acids and SLE activity, including Interleukin 6 (IL-6), high-density lipoprotein (HDL), low-density lipoprotein (LDL), cholesterol, and SLEDAI.

### 2.5. Risk of Bias Assessment

This study includes literature types of randomized controlled trials (RCTs) and case–control studies, employing different standards and methods for assessing the risk of bias for each study type. For RCTs, the risk of bias assessment is primarily conducted using standardized tools such as the Cochrane Risk of Bias Tool (RoB 1) [30]. This tool comprehensively evaluates several aspects, including bias from the random sequence generation, allocation concealment, blinding of participants and personnel, blinding of outcome assessment, incomplete outcome data, selective reporting of outcomes, and other potential sources of bias. Such a comprehensive evaluation ensures a thorough understanding and accurate assessment of the risk of bias in RCT studies. For case–control studies, the risk of bias is assessed using the Newcastle–Ottawa Scale (NOS) [31]. This scale is specifically designed for evaluating observational studies and provides detailed scoring criteria in three key areas: selection, comparability, and outcome. In the selection category, the assessment focuses on the accuracy of case definitions, the representativeness of cases and controls, and the selection criteria for controls. In the comparability category, the emphasis is on the study’s strategies for controlling confounding factors. In the outcome category, attention is given to the methods of outcome assessment and the length of follow-up for the study. These detailed scoring criteria enable a comprehensive and systematic evaluation of the quality of case–control studies.

### 2.6. Statistical Analysis

The statistical processing in this systematic review and meta-analysis was conducted using Review Manager 5.4 software. For continuous variables, we employed Standardized Mean Difference (SMD) as the effect size and reported the corresponding 95% Confidence Interval (CI). To combine the study results and assess their significance, we utilized Fisher’s Z transformation method and provided 95% CIs for the pooled effect sizes. Furthermore, to quantify heterogeneity across included studies, we employed the *I*^2^ statistic for evaluation. Depending on the degree of detected heterogeneity, we selected either a random-effects model *(I*^2^ > 50%) or a fixed-effects model (*I*^2^ < 50%) for conducting the meta-analysis. Sensitivity analyses are performed on studies with significant heterogeneity. The statistical significance of the overall effect was assessed using a Z-test, with a threshold set at *p* < 0.05. Begg’s test, Egger’s test, and funnel plotting were performed based on the number of included studies.

## 3. Results

### 3.1. Selection Criteria and Baseline Characteristics of Included Studies

**Figure 1:**
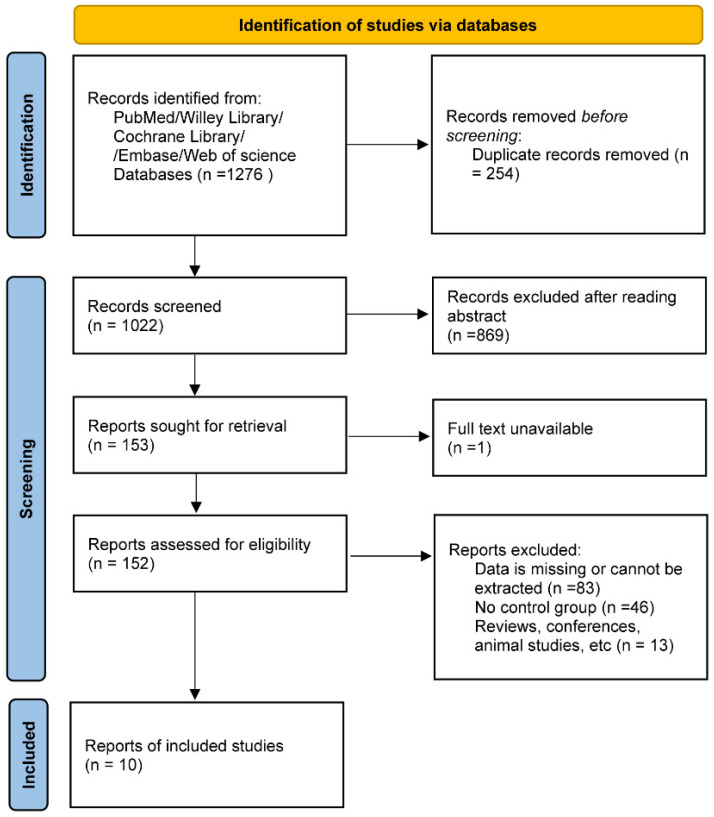
Diagram of literature selection procedure.

In our initial literature search across various databases, we identified 1276 articles. After a detailed review of titles and abstracts, 1124 articles were excluded due to duplicates, case reports, reviews, basic research, and non-controlled studies. The subsequent screening phase involved examining the full texts of the remaining 152 articles. After a thorough review, an additional 142 articles were excluded. Ultimately, our systematic review and meta-analysis included a total of 10 studies (see Figure 1). Table 1 lists the main characteristics of these studies.

### 3.2. Methodological Quality and Impact

In this study, we employed two assessment tools for evaluating the risk of bias: Rob1 and NOS, with detailed information provided in Supplementary Table S1. Using the Rob1 tool, we assessed the methodological quality of RCTs. The assessment results indicated that the majority of the included RCT studies exhibited low-to-moderate risk of bias (see Figures S1 and S2), reflecting their relatively high methodological quality. Concurrently, we utilized the NOS to evaluate case–control studies. The results showed that the scores of the included case–control studies on the NOS were generally above six (see Table S1), indicating that the selected literature met the quality standards set for this study.

### 3.3. Data Synthesis

#### 3.3.1. Relationship between Unsaturated Fatty Acid Diets and SLE Activity

**Figure 2:**
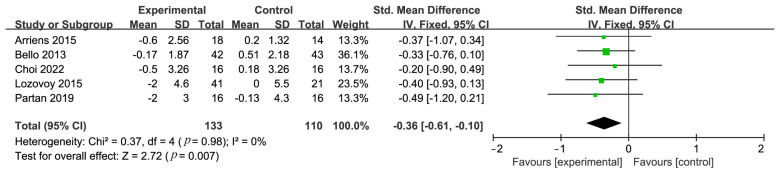
Forest plot depicting the link of unsaturated fatty acid diets with SLEDAI scores [19,21,23,32,34].

**Figure 3:**
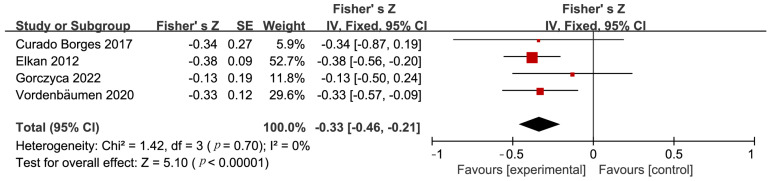
Forest plot illustrating the link of unsaturated fatty acid diets with SLE activity [20,22,24,33].

In five studies conducted on patients with SLE, researchers thoroughly investigated the potential effects of a diet rich in unsaturated fatty acids on the SLEDAI scores. The meta-analysis revealed a significant correlation between a diet high in unsaturated fatty acids and a decrease in SLEDAI scores, with a pooled SMD of −0.36, (95% CI: −0.61 to −0.11), indicating statistical significance (*p* = 0.007) (Figure 2). This finding supports the positive role of a diet rich in unsaturated fatty acids in reducing the activity of SLE disease. Furthermore, four additional studies concentrated on the link between unsaturated fatty acid diets and SLE activity. The collective results from these studies demonstrated a significant negative correlation between unsaturated fatty acid diets and the reduction of SLE activity, with a combined Fisher’s z effect size of −0.33 (95% CI: −0.46 to −0.21), *p* < 0.00001 (Figure 3), suggesting that compared to a conventional diet, a diet rich in unsaturated fatty acids significantly reduces the activity of SLE. Notably, the heterogeneity among these studies was very low (*I*^2^ = 0%, *p* = 0.70), indicating a high level of consistency and reliability in the conclusions.

#### 3.3.2. Relationship between Unsaturated Fatty Acid Diets and Inflammatory

**Figure 4:**
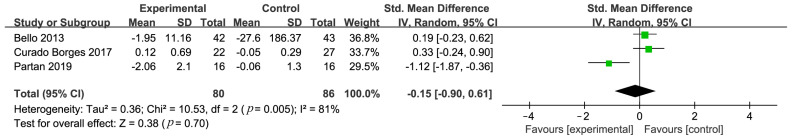
Forest plot illustrating the link between unsaturated fatty acid diets and IL-6 levels [19,23,24].

Three studies investigated the relationship between a diet rich in unsaturated fatty acids and levels of inflammatory markers. Heterogeneity analysis indicated a high level of variability among the studies (*I*^2^ = 81%), suggesting significant heterogeneity. Using a random-effects model, the meta-analysis results showed no significant difference between the diet rich in unsaturated fatty acids and the inflammatory marker Interleukin-6 (IL-6), with an SMD effect size of −0.15 (95% CI: −0.90 to 0.61), *p* = 0.70 (Figure 4).

#### 3.3.3. Relationship between Unsaturated Fatty Acid Diets and Blood Lipids

**Figure 5:**
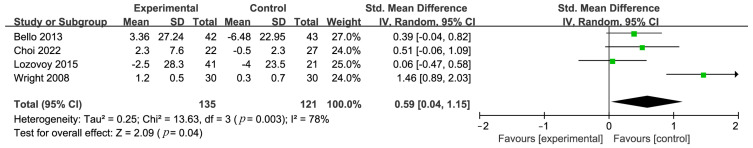
Forest plot illustrating the relationship between unsaturated fatty acid diets and HDL [21,23,34,35].

Four studies investigated the relationship between a diet rich in unsaturated fatty acids and HDL levels. Heterogeneity analysis revealed a high degree of variability among the included studies (*I*^2^ = 78%), indicating significant heterogeneity. Using a random-effects model, the meta-analysis results showed that a diet rich in unsaturated fatty acids can decrease HDL levels in patients with SLE, with an SMD effect size of 0.59 (95% CI: 0.04 to 1.15), *p* = 0.04 (Figure 5). Although the *p*-value indicates statistical significance, it is essential to consider the clinical relevance of the effect size. An SMD of 0.59 suggests a moderate effect, but the practical implications for patient health and HDL functionality are in need of further investigation.

**Figure 6:**
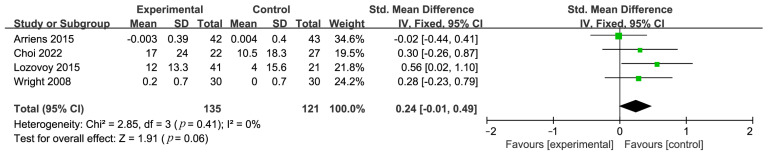
Forest plot illustrating link between unsaturated fatty acid diets and LDL [21,32,34,35].

Four studies examined the relationship between a diet rich in unsaturated fatty acids and LDL levels. Heterogeneity analysis showed a low degree of variability among the included studies (*I*^2^ = 0%), indicating low heterogeneity. Using a fixed-effects model, the meta-analysis results revealed that a diet rich in unsaturated fatty acids has no effect on LDL levels in patients with SLE, with an SMD effect size of 0.24 (95% CI: −0.01 to 0.49), *p* = 0.06 (Figure 6).

**Figure 7:**
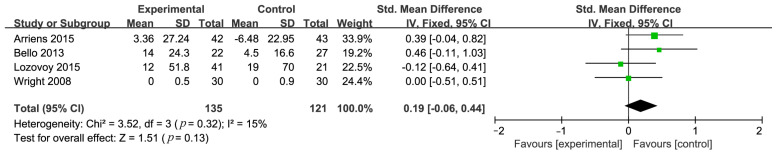
Forest plot illustrating link between unsaturated fatty acid diets and cholesterol [23,32,34,35].

Four studies investigated the relationship between a diet rich in unsaturated fatty acids and cholesterol levels. Heterogeneity analysis indicated a low degree of variability among the included studies (*I*^2^ = 15%), suggesting low heterogeneity. Using a fixed-effects model, the meta-analysis results showed that a diet rich in unsaturated fatty acids has no effect on cholesterol levels in patients with SLE, with an SMD effect size of 0.19 (95% CI: −0.06 to 0.44), *p* = 0.13 (Figure 7).

### 3.4. Sensitivity Analysis

During the sensitivity analysis aimed at evaluating the impact of the included studies on the overall meta-analysis results, re-analysis was performed after sequential exclusion of each study. The results demonstrated that the overall effect size and direction remained stable. This indicates that the overall conclusions are not significantly affected by the exclusion of any single study, thereby proving the robustness and reliability of the meta-analysis findings.

## 4. Discussion

This systematic review and meta-analysis revealed the significant potential of unsaturated fatty acids in the management of SLE, particularly with regard to regulating disease activity and improving lipid profiles. UFAs, especially omega-3 and omega-6 polyunsaturated fatty acids (PUFAs), are known for their anti-inflammatory properties, which may help suppress SLE disease activity [15,36]. Our findings align with previous studies showing that omega-3 fatty acids have beneficial effects on various rheumatic diseases, including SLE [37,38]. Building on this, our analysis further confirms the negative correlation between unsaturated fatty acid intake and the SLEDAI scores [23,24,32,34]. Previous systematic reviews have highlighted the effects of omega-3 fatty acids in various rheumatic diseases, suggesting that omega-3 fatty acids can influence disease activity by reducing the production of inflammatory mediators and improving the clinical symptoms of SLE [39,40]. Our study, through quantitative synthesis of effects on SLE activity scores and correlations, further validates the negative relationship between unsaturated fatty acid diets and SLE activity. Our research also discovered a positive impact of unsaturated fatty acids on HDL levels, while similar effects were not observed on other lipid components or the inflammatory marker IL-6. This specificity might reflect the unique mechanisms of action of unsaturated fatty acids, possibly functioning by improving vascular function, reducing blood pressure, and altering the composition and function of lipid particles [19,23,24]. For instance, omega-3 fatty acids have been shown to increase the quantity and functionality of HDL, which is associated with reduced cardiovascular disease risk [41,42]. The enhancement of HDL levels by omega-3 fatty acids can be attributed to several biological mechanisms. One such mechanism is the increased expression of apolipoprotein A-I (ApoA-I), the major protein component of HDL, which plays a critical role in HDL particle formation and cholesterol efflux from peripheral tissues to the liver for excretion [43]. This process, known as reverse cholesterol transport, is essential for maintaining lipid homeostasis and reducing the risk of atherosclerosis [44]. In addition to their effects on ApoA-I, omega-3 fatty acids can improve HDL functionality by enhancing the activity of enzymes associated with HDL metabolism, such as lecithin–cholesterol acyltransferase (LCAT) [45]. LCAT is responsible for the esterification of free cholesterol on HDL particles, which is a key step in the maturation and function of HDL [46]. Enhanced LCAT activity leads to more effective cholesterol efflux and improved HDL-mediated protection against cardiovascular diseases.

Additionally, omega-3 fatty acids can reduce inflammation by inhibiting the Nuclear Factor-kappa B (NF-κB) pathway and lowering the expression of pro-inflammatory cytokines such as Tumor Necrosis Factor-alpha (TNF-α) and IL-6 [47]. The existence of these mechanisms suggests that unsaturated fatty acids might influence the course of SLE through various pathways, including direct anti-inflammatory effects and reducing cardiovascular risk by improving the lipid profile. The role of unsaturated fatty acids in increasing HDL levels may involve other biological pathways. For instance, studies have shown that higher HDL levels can enhance its antioxidant and anti-inflammatory capabilities, further promoting vascular endothelial function improvement [48]. This offers a dual benefit for SLE patients, as it may help alleviate the chronic inflammation and associated vascular damage caused by SLE. Moreover, omega-3 fatty acids, by improving HDL functionality, enhance the activity of enzymes such as paraoxonase-1 (PON1), which provides protection against lipid oxidation [41,49]. Increased PON1 activity helps to maintain the integrity of HDL particles, making them more effective in combating oxidative stress and inflammation [50]. Another key mechanism is the modulation of lipid metabolism. Omega-3 fatty acids can increase the expression of genes involved in fatty acid oxidation and reduce the expression of genes associated with lipid synthesis, leading to improved lipid profiles [51]. This metabolic shift not only helps reduce triglyceride levels but also contributes to the increased formation and function of HDL particles. Our understanding of these mechanisms remains limited, necessitating further laboratory and clinical studies to elucidate further details. In particular, future research should explore the optimal types and dosages of unsaturated fatty acids in SLE management and how they interact with other treatment methods for SLE.

However, the role of omega-6 fatty acids remains controversial. While an adequate amount of omega-6 fatty acids is essential for normal cellular function and immune response, excessive intake may have pro-inflammatory effects, potentially negating their benefits [52]. Notably, the ratio of omega-3 to omega-6 fatty acids has been declining in recent years, a trend that appears to affect the quality of nutritional habits and their related health effects [18,53]. Studies have shown that higher omega-6 intake relative to omega-3 is associated with increased inflammation and chronic disease risk [54]. Additionally, research indicates that higher dietary omega-3 fatty acid intake and a lower omega-6 ratio are associated with better patient-reported SLE outcomes, particularly self-reported lupus activity and sleep quality [55]. Therefore, further research into the specific roles of omega-6 fatty acids and the optimal omega-6 ratio in SLE management is significant.

Despite these promising findings, the inherent limitations of the studies must be acknowledged. Although the research included studies of various designs and populations, and the included populations had similar average ages and proportions of female participants, the number of studies incorporated remains relatively small. Additionally, the differences in the types and amounts of unsaturated fatty acids used in the interventions among the included studies may affect the interpretation of the results, indicating the need for further research to identify the optimal dietary patterns beneficial for SLE patients. Furthermore, the mechanisms by which unsaturated fatty acids affect SLE disease activity and HDL levels need to be fully elucidated. Understanding these mechanisms is crucial for refining dietary recommendations and integrating them into a comprehensive treatment plan for SLE patients.

## 5. Conclusions

This systematic review and meta-analysis demonstrate that a diet rich in unsaturated fatty acids has positive effects on the management of SLE, particularly with regard to reducing disease activity and improving HDL levels. Although the impact on inflammatory markers and other lipid components is not significant, unsaturated fatty acids may provide dual benefits for SLE patients through their anti-inflammatory properties and cardiovascular health improvement. These findings support the inclusion of unsaturated fatty acids in the dietary management of SLE patients as a complement to existing pharmacological treatments. However, future studies should focus on determining the optimal ratios and types of omega-3 and omega-6 fatty acids for SLE patients, evaluating the long-term effects of these dietary interventions, and investigating the underlying mechanisms. Personalized dietary recommendations based on genetic, metabolic, and microbiome profiles should also be explored. On the other hand, larger, randomized controlled trials with diverse populations are necessary to confirm these benefits and establish clear dietary guidelines.

## Figures and Tables

**Table 1:** Basic information of included literature.

Studies.	Country	Study Design	Sample Size, *n*		Age (Years)		Number of Female Sex (%)		Intervention	
			Experimental	Control	Experimental	Control	Experimental	Control	Experimental	Control
Arriens 2015 [32]	USA	RCT	18	16	35.6 (26.3–42.7)	46.2 (36.8–46.1)	78%	79%	Fish oil	Placebo
Bello 2013 [23]	USA	RCT	42	43	45.5 ± 10.8	48.9 ± 10.6	97.60%	90.7%	Omega-3	Placebo
Choi 2022 [21]	USA	Case-Control	16	16	43.26 ± 10.59	43.97 ± 10.81	100%	100%	Fish Oil	Placebo
Curado Borges 2017 [24]	Brazil	RCT	22	27	37 (29–48)	37 (29–48)	100%	100%	Omega-3	Placebo
Elkan 2012 [33]	Sweden	Case-Control	114	122	47.9 (45.5–50.4)	49.1 (46.8–51.4)	88%	89%	Omega-3, omega-6, DHA, EPA	Placebo
Gorczyca 2022 [22]	Poland	Case-Control	30	20	47 ± 14	47 ± 14	97%	97%	*n*-3 and *n*-6 Polyunsaturated fatty acids	Placebo
Lozovoy 2015 [34]	Brazil	Case-Control	41	21	43 (32.0–51.0)	42.5 (34.0–60.0)	86%	95.20%	Fish Oil *n*-3 Fatty Acids	Placebo
Partan 2019 [19]	Indonesia	RCT	16	16	30.31 ± 9.32	26.75 ± 8.96	/	/	Seluang Fish Oil	Placebo
Vordenbäumen 2020 [20]	Germany	Case-Control	68	68	45.7 ± 12.5	45.7 ± 12.5	88%	88%	Omega-6 and -3 fatty acids	Placebo
Wright 2008 [35]	UK	RCT	30	30	48.5 ± 9.1	47.6 ± 9.6	97%	90%	Omega-3-polyunsaturated fatty acids	Placebo

## Data Availability

The original contributions presented in the study are included in the article/Supplementary Material, further inquiries can be directed to the corresponding author.

## Supplementary Materials

The following supporting information can be downloaded at: https://www.mdpi.com/article/10.3390/nu16121974/s1. File S1: PRISMA 2020 Checklist; Table S1: Assessment of the studies’ qualities using the Newcastle-Ottawa Scale; Figure S1: Risk of bias graph in the included studies; Figure S2: Risk of bias summary in the included studies.
