# Supplementary material for: Association between Unsaturated Fatty Acid-Type Diet and Systemic Lupus Erythematosus: A Systematic Review with Meta-Analyses
Source: Nutrients. 2024 Jun 20;16(12):1974. doi: 10.3390/nu16121974 (PMC11206385; doi:10.3390/nu16121974)
Supplement: Supplementary file 1 [file nutrients-16-01974-s001.zip › nutrients-3048058-supplementary/Supplementary material.pdf]

Supplementary material

Association between unsaturated fatty acid type diet and systemic lupus erythematosus: A systematic review with meta-analyses

1. Search strategy（Take PubMed search as an example）

("unsaturated fatty acids"[MeSH Terms] OR "unsaturated fatty acids"[All Fields] OR "monounsaturated fatty acids"[All Fields] OR "polyunsaturated fatty acids"[All Fields] OR "omega-3 fatty acids"[All Fields] OR "omega-6 fatty acids"[All Fields] OR "fish oil"[All Fields]) AND ("diet"[MeSH Terms] OR "dietary intake"[All Fields] OR "diet"[All Fields]) AND ("systemic lupus erythematosus"[MeSH Terms] OR "systemic lupus erythematosus"[All Fields] OR "SLE"[All Fields] OR "autoimmune diseases"[MeSH Terms] OR "autoimmune diseases"[All Fields])

2. Risk of bias in the included studies

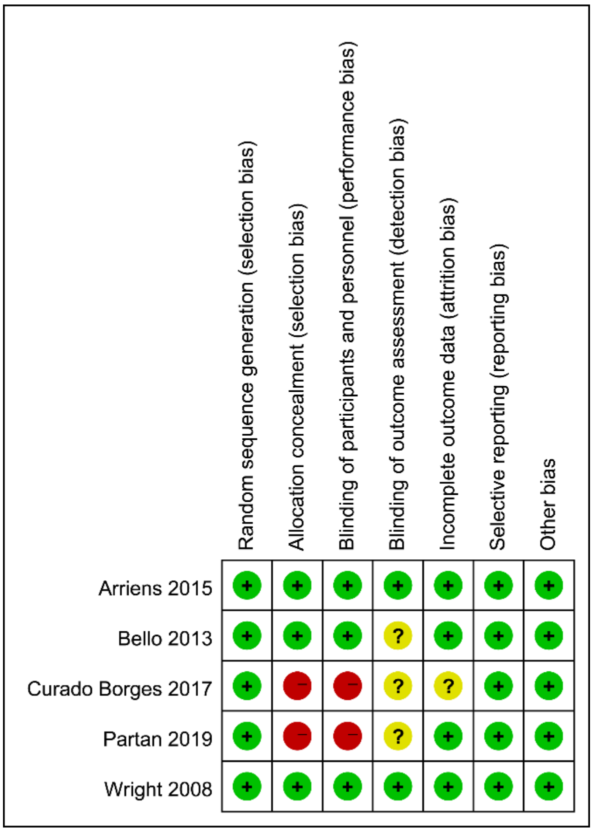

Figure S1. Risk of bias graph in the included studies

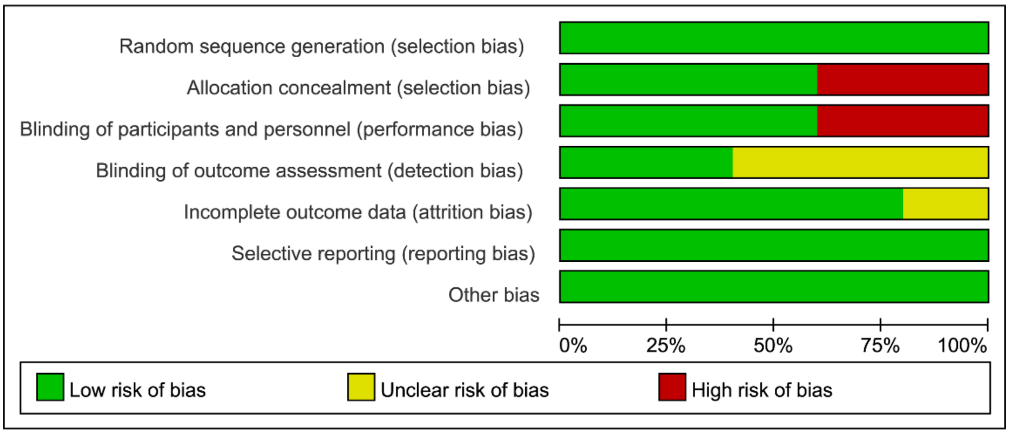

Figure S2. Risk of bias summary in the included studies

Table S1. Assessment of the studies' qualities using the Newcastle-Ottawa Scale.

Authors	Year	Region	Selection	Comparability	Exposure	Quality Score
Choi 2022	2022	USA	★★★	★★	★★★	★★★★★★★★
Elkan 2012	2012	Sweden	★★★	★	★★	★★★★★★
Gorczyca 2022	2022	Poland	★★★	★	★★	★★★★★★
Lozovoy 2015	2015	Brazil	★★★★	★★	★★	★★★★★★★★
Vordenbäumen 2020	2020	Germany	★★★	★	★★	★★★★★★

Each study included was judged in three broad categories by using the "star system": the selection of study groups, the comparability of their cases and controls, and the ascertainment of exposure for cases and control.
